# The Drug Developments of Hydrogen Sulfide on Cardiovascular Disease

**DOI:** 10.1155/2018/4010395

**Published:** 2018-07-29

**Authors:** Ya-Dan Wen, Hong Wang, Yi-Zhun Zhu

**Affiliations:** ^1^Department of Clinical Pharmacology, Xiangya Hospital, Central South University, Changsha, China; ^2^Hunan Key Laboratory of Pharmacogenetics, Institute of Clinical Pharmacology, Central South University, Changsha, China; ^3^Department of Pharmacology, Yong Loo Lin School of Medicine, National University of Singapore, Singapore; ^4^Singapore Nuclear Research and Safety Initiative, National University of Singapore, Singapore; ^5^Department of Pharmacology, Macau University of Science and Technology, Macau

## Abstract

The recognition of hydrogen sulfide (H_2_S) has been evolved from a toxic gas to a physiological mediator, exhibiting properties similar to NO and CO. On the one hand, H_2_S is produced from L-cysteine by enzymes of cystathionine *γ*-lyase (CSE) and cystathionine *β*-synthase (CBS), 3-mercaptopyruvate sulfurtransferase (3MST) in combination with aspartate aminotransferase (AAT) (also called as cysteine aminotransferase, CAT); on the other hand, H_2_S is produced from D-cysteine by enzymes of D-amino acid oxidase (DAO). Besides sulfide salt, several sulfide-releasing compounds have been synthesized, including organosulfur compounds, Lawesson's reagent and analogs, and plant-derived natural products. Based on garlic extractions, we synthesized S-propargyl-L-cysteine (SPRC) and its analogs to contribute our endeavors on drug development of sulfide-containing compounds. A multitude of evidences has presented H_2_S is widely involved in the roles of physiological and pathological process, including hypertension, atherosclerosis, angiogenesis, and myocardial infarcts. This review summarizes current sulfide compounds, available H_2_S measurements, and potential molecular mechanisms involved in cardioprotections to help researchers develop further applications and therapeutically drugs.

## 1. Introduction

In an evolutionary perspective, the synthesis and catabolism of hydrogen sulfide (H_2_S) by living organisms antedates the evolution of vertebrate. Bacteria and archaea produce and utilize the stinking gas as one of the essential sources for their survival and proliferation. For many decades, H_2_S, the colorless gas with a strong odor of rotten gas, is recognized as a toxic gas and an environmental pollutant. The mechanism of its toxicity is a potent inhibition of mitochondrial cytochrome c oxidase, which is the important enzyme that is closely related with chemical energy in the form of adenosine triphosphate (ATP). Sulfide, together with cyanide, azide, and carbon monoxide (CO), all can inhibit cytochrome c oxidase which leads to chemical asphyxiation of cells.

In the last two decades, the perception of H_2_S has been changed from that of a noxious gas to a gasotransmitter with vast potential in pharmacotherapy. At the end of the 1980s, endogenous H_2_S is found in the brain [1]. Then, its enzymatic mechanism, physiological concentrations, and specific cellular targets were described in the year 1996 [2]. Subsequently, the physiological and pharmacological characters of H_2_S were unveiled. Recently, H_2_S, followed with NO and CO, is identified as the third gasotransmitter by Wang [3]. The three gases share some common features. They are all colorless and poisonous gases. With the exception of gas pressure in atmosphere, they can dissolve in water at different solubility. All these small signaling molecules possess significant physiological importance, like anti-inflammation and antiapoptosis. The similarities and differences of the features of NO, CO, and H_2_S are summarized in Table 1.

This review is prepared for researchers, who are interested in H_2_S and sulfide-containing compounds, on drug development of cardiovascular disease. Therefore, some key issues were discussed, like “donors and inhibitors” to support choosing the sulfide-releasing chemicals and specific inhibitors. Readers could depend on the precision of currently “measuring methods” to decide the analyzing techniques. H_2_S on “inflammation,” “redox status,” and “cardiovascular disease” summarizes the currently novel findings of the effects of H_2_S and underlying mechanisms.

## 2. Physical and Biological Characteristics

H_2_S, a colorless and flammable gas with the characteristic foul odor of rotten eggs, is known for decades as a toxic gas and an environmental hazard. It is soluble in water (1 g in 242 ml at 20°C). In water or plasma, H_2_S is a weak acid which hydrolyzes to hydrogen ion and hydrosulfide and sulfide ions as following: H_2_S ↔ H^+^ + HS^−^ ↔ 2H^+^ + S^2−^. The pKa at 37°C is 6.76. When H_2_S is dissolved in physiological solution (pH 7.4, 37°C), it yields approximately 18.5% H_2_S and 81.5% hydrosulfide anion (HS^−^), as predicted by the Henderson-Hasselbalch equation [4]. H_2_S could be oxidized to sulfur oxide, sulfate, persulfide, and sulfite. H_2_S is permeable to plasma membranes as its solubility in lipophilic solvents is fivefold greater than in water. In other words, it is able to freely penetrate cells of all types.

The toxic effect of H_2_S on living organisms has been recognized for nearly 300 years, and until recently, it was believed to be a poisonous environmental pollutant with minimal physiological significance. H_2_S is more toxic than hydrogen cyanide and exposed to as little as 300 ppm in the air for just 30 min is fatal to human. The level of odor detection of sulfide by the human nose is at a concentration of 0.02–0.1 ppm, 400-fold lower than the toxic level. As a broad-spectrum toxicant, H_2_S affects many organ systems including the lung, brain, and kidney.

**Figure 1:**
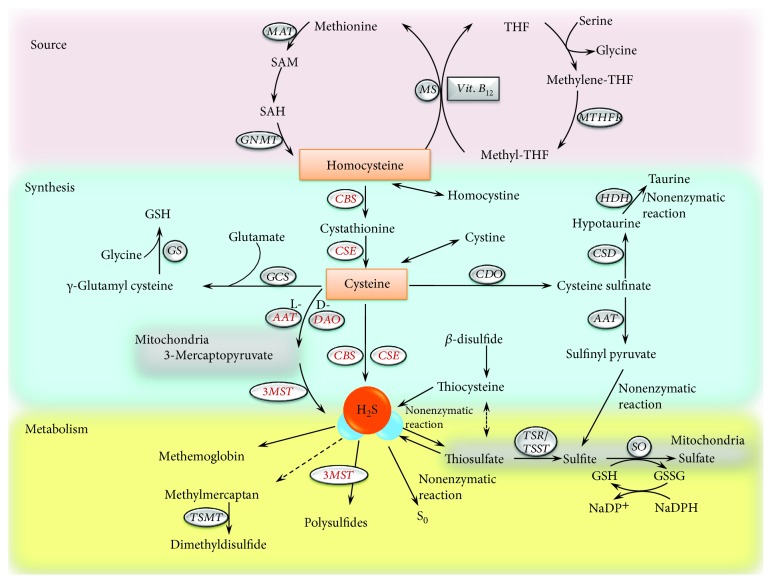
Synthesis and catabolism of H_2_S. AAT: aspartate aminotransferase; CDO: cysteine dioxygenase; CSE: cystathionine *γ*-lyase; HDH: hypotaurine dehydrogenase; GCS: *γ*-glutamyl cysteine synthase; GS: glutathione synthase; MAT: methionine adenosyltransferase; MS: methionine synthase; S0: elemental sulfur; SAM: S-adenosylmethionine; THF: tetrahydrofolate; TSST: thiosulfate sulfurtransferase; CBS: cystathionine *β*-synthase; CSD: sulfinate decarboxylase; DAO: D-amino acid oxidase; H_2_S: hydrogen sulfide; GNMT: glycine N-methyltransferase; GSH: glutathione; 3MST: 3-mercaptopyruvate sulfide transferase; MTHFR: methylenetetrahydrofolate reductase; SAH: S-adenosylhomocysteine; SO: sulfite oxidase; TSR: thiosulfate reductase; TSMT: thiol S-methyltransferase.

H_2_S is often produced through the anaerobic bacterial breakdown of organic substrates in the absence of oxygen, such as in swamps and sewers (anaerobic digestion). It also results from inorganic reactions in volcanic gases, natural gas, and some well waters. Digestion of algae, mushrooms, garlic, and onions is believed to release H_2_S by chemical transformation and enzymatic reactions [5]. Structures of natural food-releasing H_2_S on digestion are shown in Figure 1. Consuming mushrooms, garlic, and onions, which contain chemicals and enzymes responsible for the transformation of the sulfur compounds, is responsible for H_2_S production in the human gut [6]. Human body produces small amounts of H_2_S and uses it as a signaling molecule. In different species and organs, the concentration of H_2_S varies in different levels. In Wistar rats, the normal blood level of H_2_S is 10 *μ*M [7]; while in Sprague-Dawley rats, the plasma level of H_2_S increases to 46 *μ*M [8]; in human, 10–100 *μ*M H_2_S in blood was reported [9]. The tissue level of H_2_S is known to be higher than its circulating level. The concentration of endogenous H_2_S has been reported up to 50–160 *μ*M in the brains of rat, human, and bovine [1, 10, 11]. Significant amounts of H_2_S are generated from vascular tissues, and this production varies among different types of vascular tissues. For instance, the homogenates of thoracic aorta yielded more H_2_S than that of portal vein of rats [8]. Furne et al. reported that in situ tissue H_2_S level through analyzing the gas space over rapidly homogenized mouse brain and liver was only 15 nM [12].

## 3. Synthesis and Catabolism of H_2_S

H_2_S is endogenously formed by both enzymatic and nonenzymatic pathways [3]. The enzymatic procedure of synthesizing H_2_S, in mammalian tissues, is involved in two pyridoxal 5′-phosphate-dependent enzymes: cystathionine *γ*-lyase (CSE) and cystathionine *β*-synthase (CBS) [13–15]. As shown in Figure 1, H_2_S is catalyzed from the desulfhydration of L-cysteine, a sulfur containing amino acid derived from alimentary sources, produced by the transsulfuration pathway of L-methionine to homocysteine or liberated from other endogenous proteins [16, 17]. As the intermediate, CBS catalyzes homocysteine together with serine to yield cystathionine, which is converted to cysteine, *α*-ketobutyrate, and NH_4_^+^ by CSE. The two pyridoxal 5′-phosphate-dependent enzymes both or either catalyze the conversion of cysteine to H_2_S, pyruvate, and NH_4_^+^. CSE also could catalyze a *β*-disulfide elimination reaction that results in the production of thiocysteine, pyruvate, and NH_4_^+^. Thiocysteine is associated with cysteine or other thiols to form H_2_S [18]. The two synthesis pathways of producing H_2_S are illustrated in Figure 1.

The two enzymes are widespread in mammalian tissues and cells and also in many invertebrates and bacteria [19]. The activity of CSE is chiefly concentrated in the liver, heart, vessels, kidney, brain, small intestine, stomach, uterus, placenta, and pancreatic islets; whereas, the amount of CBS is mainly located in the brain, liver, kidney and ileum, uterus, placenta, and pancreatic islets [20]. The locations of H_2_S-producing enzymes are seen in Table 2. In several species, the liver is the common organ containing the two enzymes in abundance. According to the research of Zhao et al., the intensity rank of biosynthesis of H_2_S by origin of exogenous cysteine in different rat blood vessels was tail artery > aorta > mesenteric artery [21].

A third enzymatic reaction contributing to H_2_S production has recently been identified in brain and vascular endothelium, that is, 3-mercaptopyruvate sulfurtransferase (3MST) in combination with aspartate aminotransferase (AAT) (also called cysteine aminotransferase, CAT) [22, 23], seen in Figure 1. In mitochondria, L-cysteine and *α*-ketoglutarate as substrates can be converted to 3-mercaptopyruvate (3MP) by AAT; then, the intermediate product is converted to H_2_S by 3MST [23]. In the brain, 3MST is found in neurons [24] and astrocytes [25], while CBS in astrocytes [24]. It could speculate that the two enzymes of catalyzing H_2_S play different roles in the nervous system. In vascular tissues, 3MST could be detected in both endothelial cells and vascular smooth muscle cells (SMCs), while AAT just occurs in endothelial cells. From another perspective, only vascular endothelial cells in vessel could utilize the two enzymes to produce H_2_S, whereas vascular SMCs likely absorb 3-mercaptopyruvate or other sources to generate H_2_S which exerts as a vasodilator.

The fourth enzymatic pathway was recently reported by Shibuya et al. [26] that produces H_2_S from D-cysteine by D-amino acid oxidase (DAO). Different from using L-cysteine to produce H_2_S by CBS, CSE, and 3MST/AAT, which are pyridoxal 5′-phosphate- (PLP-) dependent enzymes, D-cysteine pathway generates H_2_S by PLP-independent enzyme [27]. Similar to 3MST on mitochondria, DAO localizes to peroxisomes in mitochondrial fractions [28]. D-cysteine is metabolized by DAO in peroxisomes to achiral 3MP, which is also generated from L-cysteine by AAT [27, 29]. 3MP then is metabolized to final H_2_S through 3MST, due to the vesicular trafficking between mitochondria and peroxisomes [30]. The key enzyme in new D-cysteine pathway, DAO was verified by DAO-selective antagonist I2CA, which suppressed the production of 3MP and H_2_S from D-cysteine in concentration-dependent manner, but that from L-cysteine was not influenced by I2CA [26]. This new enzymatic H_2_S-producing pathway is integrated into the part of “synthesis” in Figure 1.

The nonenzymatic route of yielding H_2_S is the conversion of elemental sulfur and transformation of oxidation of glucose. The nonenzymatic route is presented in vivo, involving phosphogluconate (<10%), glycolysis (>90%), and glutathione (<5%) [3].

In the pathway of H_2_S production, there are several important amino acids: homocysteine and D-cysteine. Besides the generation of H_2_S pathway, homocysteine is related to folate cycle and methionine cycle [31], the latter of which is participated in methionine, SAM and SAH, as previously stated. As the bridge of the two cycles, homocysteine could be remethylated to methionine by interacting with methylenetetrahydrofolate (methyl-THF) and vitamin B_12_ as cofactor under the synthesis of methionine synthase (MS). Methyl-THF is transformed from methylenetetrahydrofolate (methylene-THF) by methylenetetrahydrofolate reductase (MTHFR). Tetrahydrofolate (THF) is generated by remethylation and converted to methylene-THF, thus integrated the folate cycle. In another cycle, methionine is transformed to S-adenosylmethionine (SAM) by methionine adenosyltransferase (MAT) and then is converted to S-adenosylhomocysteine (SAH), which is subsequently hydrolyzed to homocysteine by glycine N-methyltransferase (GNMT). The cycles of homocysteine can assist researchers to link the studies of upstream and downstream of H_2_S, as illustrated in Figure 1. The second interesting amino acid is D-cysteine, because mammalian enzymes generally metabolize L-amino acids, except a little few like D-aspartate and D-serine [29]. Previously, D-cysteine is widely used as a negative control for L-cysteine until discovered as a highly effective H_2_S-producing source by Hideo group [26]. As the key enzymes in D-cysteine pathway, DAO is localized in the cerebellum and kidney, together with 3MST [26]. After birth, the level of DAO increased then reached maximal at 8 weeks in mice, while the level of 3MST was quite high at birth but slightly reduced at 8 weeks in mice [27]. Taken together, the level of H_2_S through D-cysteine pathway rose after birth and rocketed to maximal at 6 weeks [27]. The level of H_2_S generated from L-cysteine was much lower than that from D-cysteine and remains in a certain amount over time. Additionally, the generation of H_2_S from D-cysteine is 80 times more efficient than that from L-cysteine in the kidney [26]. Moreover, the generation of H_2_S from D-cysteine in the kidney is 7 times higher than that in the cerebellum, which is the region producing highest level of H_2_S from D-cysteine than other parts in the brain [26]. Since H_2_S has presented significant therapeutic potentials on anti-inflammation, antioxidation, antiapoptosis, antimitochondrial dysfunction, and energy reservation, the new D-cysteine pathway in the kidney and cerebellum may provide researchers new ideas of finding therapeutic approaches on brain and kidney diseases, such as kidney transplantation.

Cysteine metabolism is engaged in three major routes. Apart from the conversion of H_2_S, one path is oxidation of -SH group by cysteine dioxygenase (CDO) to cysteine sulfinate, which is decarboxylated to hypotaurine by cysteine sulfinate decarboxylase (CSD) and then further transformed to taurine by a nonenzymatic reaction or by hypotaurine dehydrogenase (HDH) or which is converted to sulfinyl pyruvate, subsequently to sulfite and further sulfate. Another path from cysteine is synthesis GSH by glutathione synthase (GS) from *γ*-glutamyl cysteine, which is originated from cysteine and glutamate catalyzed by *γ*-glutamyl cysteine synthase (GCS). Besides H_2_S, cysteine metabolism is integrated in Figure 1 for helping researchers to find out the potential associations.

The concentration of H_2_S is not only determined by the rate of formation but also by degradation of H_2_S. Dissolved gaseous H_2_S is in a pH-dependent equilibrium, with hydrosulfide anions (HS^−^) and sulfide anions (S^2−^), which can be catabolized to any sulfur-containing molecule. Sulfide, via nonenzymatic route, is catabolized to thiosulfate, which could be catalyzed to sulfite by thiosulfate reductase (TSR) in the livers, brains, or kidneys, or by thiosulfate sulfurtransferase (TSST) in the livers, sequentially oxidized to sulfate via sulfite oxidase (SO) by a glutathione- (GSH-) dependent reaction. The last product is excreted in urine [32]. H_2_S could be broken down by rhodanese, methylated to CH_3_SH, sequestrated by methemoglobin, interacted with superoxide or NO, and scavenged by metallo- or disulfide-containing molecules such as oxidized glutathione [18, 19]. The major routes of degradation of H_2_S through nonenzymatic oxidation of sulfide also yield elemental sulfur, polysulfides, dithionate, and polythionates. Among them, polysulfides could be produced through the enzymatic way via 3MST [33–35] and the chemical interaction of H_2_S with NO [36]. The whole schematic version of source, synthesis, and metabolism of H_2_S is depicted in Figure 1.

## 4. Donors and Inhibitors of H_2_S

### 4.1. The Donors of H_2_S

#### 4.1.1. Sulfide-Containing Salts

Sodium hydrogen sulfide (NaHS) and disodium sulfide (Na_2_S) are the common H_2_S-releasing chemicals in research of hydrogen sulfide. These sodium salts purchased from pharmaceutical companies are usually aquo compounds, like NaHS·12H_2_O, Na_2_S·9H_2_O, or anhydrous forms. The products of sodium hydrogen sulfide and disodium sulfide should be white. The pills with yellow color predicate the anhydrous forms have been converted to hygroscopic blocks and should not be purchased. White sulfide products are likely to have greater purity, but may contain sodium salts of thiosulfate or higher oxidation state sulfur oxyanions [37]. Contamination by trace metal ions may also be important, as these catalyze oxidation processes. The sulfides should therefore be reserved in a vacuum desiccator to minimize oxidation.

The solution of NaHS, at physical pH and room temperature, hydrolyzes to sodium ion, hydrosulfide as following: NaHS ↔ Na^+^ + HS^−^. Solutions of HS^−^ are sensitive to oxygen, converting mainly to polysulfides, indicated by the appearance of yellow color. Hence, solutions of fresh prepared NaHS should be clear and put to use immediately. The purity of sulfides could be measured by determining the sulfide content either by titration with bromate, as described in standard analytical chemistry texts, or by UV spectroscopy in the case of sodium hydrogen sulfide, at pH 9, which has an absorption maximum at 230 nm with a molar absorptivity of 7200 l/mol/cm [38].

Considering the unstable chemical properties of NaHS and Na_2_S, some researchers introduce another donor of H_2_S, calcium sulfide (CaS), which is more steady [39]. CaS can be found as one of the effective components in a traditional herb, named “hepar sulfuris calcareum,” usually applied to homeopathic remedy. Oral administration of CaS will be decomposed to more H_2_S in stomach acid environment. This review postulates CaS may carry out hypotension, arguing from its catabolism, relationship of calcium supplementation and blood pressure, dosage design, and traditional application of homeopathic remedy on infection.

#### 4.1.2. H_2_S-Releasing Molecules

Thioacetamide is an organosulfur compound with the formula C_2_H_5_NS. This white crystalline solid is soluble in water and serves as a source of sulfide ions in the synthesis of organic and inorganic compounds [40]. For lab safety, thioacetamide is carcinogen class 2B and has hepatotoxicity. Thioacetamide was widely used in classical qualitative inorganic analysis as an in situ source for sulfide ions.

Some research laboratories developed H_2_S releasers. Lawesson's reagent is a chemical compound used in organic synthesis as a thiation agent and is also a H_2_S releaser. Lawesson's reagent is first synthesized in 1956 during a systematic study of the reactions of arenes with P_4_S_10_ [41]. After much time, it is first made popular by Sven-Olov Lawesson for introducing a thiation procedure as an example of a general synthetic method for the conversion of carbonyl to thiocarbonyl groups [41]. 2,4-Bis (4-methoxyphenyl)-1,3,2,4-dithiadiphosphetane 2,4-disulfide, Lawesson's reagent, has a four-membered ring of alternating sulfur and phosphorus atoms. Normally in higher temperatures, the central phosphorus/sulfur four-membered ring can open to form two reactive dithiophosphine ylides (R-PS2), which decompose to release H_2_S. As its strong and unpleasant smell, it is best to prepare Lawesson's reagent within a fume hood and treat all glassware used with a decontamination solution before taking the glassware outside the fume hood.

**Figure 2:**
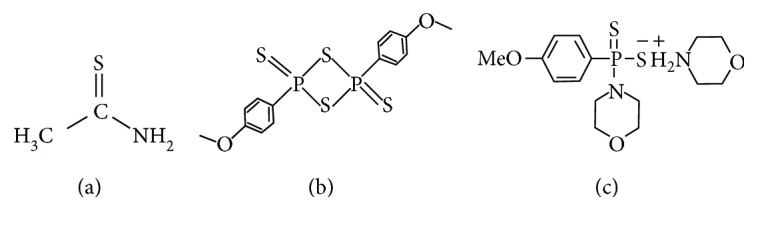
Structures of H_2_S-releasing molecules.

Based on Lawesson's compound, a series of compounds are synthesized. Professor Moore's lab reports that morpholin-4-ium-4-methoxyphenyl (morpholino) phosphinodithioate (GYY4137) releases H_2_S slowly both in vitro and in vivo. It has been proved that GYY4137 has vasodilator and antihypertensive activities and a useful H_2_S-releasing chemical in the study of biological effects of H_2_S [42]. In a later experiment, administration of GYY4137 to lipopolysaccharide- (LPS-) induced rats displays its anti-inflammatory effect by increasing plasma anti-inflammatory cytokine IL-10 and reducing plasma proinflammatory cytokines (TNF-*α*, IL-1*β*, and IL-6) and nitrite/nitrate, C-reactive protein, and L-selectin [43]. Structures of H_2_S-releasing molecules are shown in Figure 2.

Considering pharmacological effects and adverse effects of H_2_S, some pharmaceutical factories join in working on H_2_S donors which are made up of well-established parent compounds and H_2_S-releasing moieties. CTG Pharma developed ACS series H_2_S-releasing compounds to meet their interests on the aspects of hypertension, metabolic syndrome, thrombosis, and arthritis (http://www.ctgpharma.com). Antibe Therapeutics synthesizes several ATB series H_2_S-releasing derivatives for the treatments of inflammatory bowel disease, joint pain, and irritable bowel syndrome (http://www.antibe-therapeutics.com). The compound, IK-1001, from the company Ikaria, is an injectable form of Na_2_S, which is pure, pH neutral, and stable. IK-1001 has been used several basic studies and processed into clinical trials. One is a phase I safety trial for assessing pharmacokinetics of intravenous IK-1001 (ClinicalTrials.gov ID: NCT00879645). Another is a phase II efficacy trial which administers IK-1001 in patients undergoing surgery for a coronary artery bypass graft (ClinicalTrials.gov ID: NCT00858936). The effects of some H_2_S-releasing compounds are shown in Table 3.

#### 4.1.3. Natural Products Containing Sulfur

**Figure 3:**
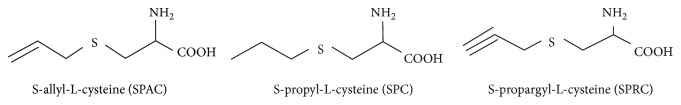
The chemical structures of SAC, SPC, and SPRC.

Digestion of algae, mushrooms, garlic, and onions is believed to form H_2_S by chemical transformation and enzymatic reactions [5]. Structures of natural food-releasing H_2_S on digestion are shown in Figures 2 and 3. Nearly all the allium families are sulfur-rich containing. Several publication reports enumerated functional activities of garlic. It exhibits hypolipidemic, antimicrobial, antiplatelet, and procirculatory effects [44–46]. It also demonstrates immune enhancement and provides anticancer, antimutagenic, and antiproliferative that are interesting in chemopreventive interventions. Additionally, aged garlic extract possesses hepatoprotective, neuroprotective, and antioxidative activities [47]. The major sulfur-containing compounds in intact garlic are *γ*-glutamyl-S-allyl-L-cysteines and S-allyl-L-cysteine sulfoxides (alliin). Both are abundant as sulfur compounds, and alliin is the primary odorless, sulfur-containing amino acid, a precursor of allicin, methiin, (+)-S-(trans-1-propenyl)-L-cysteine sulfoxide, and cycloalliin [48].

S-allylcysteine (SAC), a major transformed product from *γ*-glutamyl-S-allyl-L-cysteine, is a sulfur amino acid detected in the blood that is verified as both biologically active and bioavailable [49], as seen in Figure 3. SAC has been enumerated in several research investigations mediating protective effects in neural system and cardiovascular system by the inhibition of cell damage in the neuron, heart, and endothelium. In neural system, it is reported that SAC may attenuate A*β*-induced apoptosis [50] and destabilize Alzheimer's A*β* fibrils in vitro [51]. SAC prohibits cerebral amyloid, cerebral inflammation, and tau phosphorylation in Alzheimer's transgenic mouse model harboring Swedish double mutation [52]. In stroke-prone spontaneously hypertensive rats, intaking SAC diminishes incidence of stroke, impairs behavioral syndromes, and abates mortality induced by stroke [53]. SAC inhibits free radical production, lipid peroxidation, and neuronal damage in rat brain ischemia [54]. In cardiovascular system, SAC can help the acute myocardial infarction rats survived by significantly lowering mortality and reducing infarct size [55].

S-propyl-L-cysteine (SPC) and S-propargyl-L-cysteine (SPRC) are structural analogues of SAC, differing only in the propargyl and allyl moiety, respectively, while containing the same cysteine structure as shown in Figure 3. Wang et al., from our lab, reported that SPRC exhibited stronger cardioprotective effects than SAC in reducing mortality, increasing cell viability, reducing heart infarct size, lowering LDH and CK levels and activities, and having antioxidant properties [56]. These data suggest that the propargyl group of SPRC further increases the affinity and/or activity of SPRC towards the enzyme CSE as compared to SAC, where SPRC treatment is shown to have an increased CSE expression and activity to produce H_2_S for coping with ischemic damage. This observation suggests that the cardioprotective effects involving the CSE/H_2_S pathway were more effective using SPRC compared to SAC. Recently, our lab reported that SPRC showed neuroprotective effects of cognitive impairment and inhibition of neuronal ultrastructure damage in A*β*-induced rats, affords a beneficial action on anti-inflammatory pathways [57]. SPRC has been demonstrated the anticancer effect on gastric cancer at high doses 50 mg/kg/d and 100 mg/kg/d [58]. The effects of SAC and SPRC are shown in Table 3.

### 4.2. The Inhibitors and Regulators of H_2_S

The production of H_2_S from cysteine by tissue/cell homogenate is decreased by the presence of inhibitors of H_2_S-producing enzymes, which are mainly attributed to CSE and CBS. CSE is also named as cysteine desulfhydrase [59]. The CBS locus is mapped to chromosome 21 (21q22.3) [60]. Several specific blockers for CSE and CBS are currently available. D,L-Propargylglycine (PAG) and b-cyano-L-alanine selectively inhibit CSE [8]. L-Cysteine metabolites, including ammonia, H_2_S, and pyruvate, cannot inhibit CSE activity [61]. CBS is inhibited by hydroxylamine (HA) and aminooxyacetate (AOAA) albeit these chemicals are not selective inhibitors of CBS [2]. The relationships between H_2_S-producing enzymes and their inhibitors are summarized in Table 2.

The currently known regulations of H_2_S-producing enzymes are glutamate and its receptors, S-adenosyl-methionine (SAM), hormones, and other gasotransmitters—NO and CO. In the brain, electrical stimulation and excitatory neurotransmitter, glutamate, rapidly increase CBS activity in Ca^2+^/calmodulin-dependent manner [62]. Both *α*-amino-3-hydroxy-5-methyl-4-isoxazolepropionate (AMPA) glutamate receptors and N-methyl-D-aspartate (NMDA) are involved in this effect. SAM is an intermediate product of methionine metabolism and a major donor of methyl groups. This allosteric regulator can activate CBS by approximately twofold [2]. Sex hormones seem to regulate brain H_2_S, since CBS activity and H_2_S level are higher in male than in female mice and castration of male mice decreases H_2_S formation [16]. Sodium nitroprusside, a nitric oxide donor, increases the activity of brain CBS *in vitro*; however, this effect is NO-independent and results from chemical modification of the enzyme's cysteine groups [63]. In contrast, NO itself may bind to and inactivate the CBS. Interestingly, CO is a much more potent CBS inhibitor than NO and it is suggested that CBS may be one of the molecular targets for CO in the brain [64, 65]. In homogenates of the rat aorta, NO donors acutely increase CSE-dependent H_2_S generation in a cGMP-dependent manner [21]. Moreover, prolonged incubation of cultured vascular smooth muscle cells in the presence of NO donors increases CSE mRNA and protein levels [8]. The physiological significance of NO in the regulation of H_2_S production is also supported by the observation that circulating H_2_S level as well as CSE gene expression and enzymatic activity in the cardiovascular system are reduced in rats chronically treated with NOS inhibitor. Thus, NO is probably a physiological regulator of H_2_S production in the cardiovascular system. Recently, the inhibitors of 3MST were selected by high-throughput screening (HTS) of a large chemical library (174,118 compounds) with the H_2_S-selective fluorescent probe, HSip-1, which discovered compound 3 presented very high selectivity for 3MST over other H_2_S/sulfane sulfur-producing enzymes and rhodanese [66]. This study provides these compounds as useful chemical tools for investigating the physiological roles of 3MST.

## 5. H_2_S Measurements

### 5.1. Spectrophotometric Method

**Figure 4:**
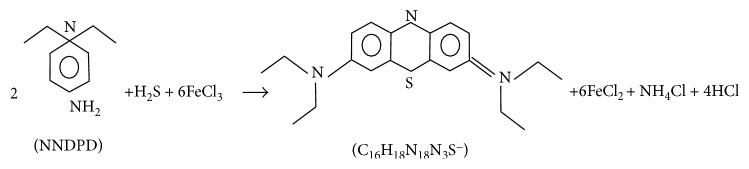
The equation of spectrophotometric method of H_2_S.

The principle of spectrophotometric method of H_2_S depends on the formation of methylene blue. H_2_S is chemiadsorbed by zinc acetate and transformed into stable zinc sulfide. The sulfide is recovered by extraction with water. In contact with an oxidizing agent such as ferric chloride in a strongly acid solution, it reacts with the N,N-dimethyl-p-phenylenediammonium (NNDPD) ion to yield methylene blue (C_16_H_18_N_3_SCl). The equation is shown in Figure 4:

The methylene blue method has been designed to a different protocol. A common method is adding NNDPD and ferric chloride to the plasma or homogenized tissue and then developing color and colorimetric estimating immediately. Owing to the volatile character of H_2_S, researchers modify the protocol, like using a filter paper to augment the contact surface and prolong the contact time [67, 68]. Based on published papers and previous experience, our lab revised the assay for H_2_S by placing a sample in an airtight vessel with a central tube. The central tube contains a filter paper wick saturated with zinc acetate. The purpose of the filter paper wick is for trapping H_2_S to zinc sulfide. The reactions are initiated by mixing of strong acid with the sample, which sulfide is driven out and adsorbed onto the wick. The driving time is usually 30–120 minutes which is modified based on lab condition and optimization in the sorts of samples. Reactions are stopped by injecting 0.5% trichloroacetic acid (TCA). After gas evolution and wick absorption, the sulfide in the central tube reacts with NNDPD in present of Fe^2+^ ion. The absorbance of the resulting solution at 670 nm was measured with a microplate reader. This method was improved by Ishigami et al. through the release of H_2_S from acid-labile sulfur using acids as an artifact, which leads H_2_S absorbed immediately and stored as bound sulfur [24].

This colorimetric method is not only widely used on the determination of H_2_S on serum in animal experiment but also widely used on the activity of CSE/CBS enzyme on tissues or cells. The concentrations of H_2_S are reflected on the different shades of color of methylene blue and calculated by the plotting H_2_S standard curve.

Two points need to be made. Firstly, most researchers' assay H_2_S using the spectrophotometric assay involves acidifying zinc acetate-treated (to “trap” free H_2_S) biological samples in the presence of a dye and observing a color change. This assay actually measures total sulfide and not the gas H_2_S. Secondly, H_2_S is either broken down rapidly in the body by enzymes, sequestered by binding to hemoglobin, or can react chemically with a number of species abundant in tissues, including superoxide radical [69], hydrogen peroxide [67], peroxynitrite [70], and/or hypochlorite [71]. All in all, making reasonably accurate measurements of such an evanescent and reactive gas in biological tissues is difficult. Indeed, the chemical nature of gases such as H_2_S, NO, and CO might render it nonsensical even to try and measure them in body fluids or tissues.

### 5.2. Sulfide Ion-Selective Electrode

A sulfide ion-selective electrode (SISE) is immersed in an aqueous solution containing the ions to be measured, together with a separate, external reference electrode. The electrochemical circuit is completed by connecting the electrodes to a sensitive millivoltmeter using special low-noise cables and connectors. A potential difference is developed across the SISE membrane when the sulfide ions diffuse through from the high concentration side to the lower concentration side.

At equilibrium, the membrane potential is mainly dependent on the concentration of the target ion outside the membrane and is described by the Nernst equation. Briefly, the measured voltage is proportional to the logarithm of the concentration, and the sensitivity of the electrode is expressed as the electrode slope in millivolts per decade of concentration. Thus, the electrodes can be calibrated by measuring the voltage in sulfide standard solution. Testing samples can then be determined by measuring the voltage and plotting the result on the calibration graph. The use of sulfide ion-selective electrode suffers from precipitation of metal sulfide, for example, sliver sulfide (Ag_2_S) from the filling solution on the electrodes.

Reproducibility is limited by factors such as temperature fluctuations, drift, and noise. The electrode can be used at temperatures from 0 to 100°C and only used intermittently at temperatures above 80°C. Interfering ions, like mercury, must be absent from all sulfide sample. In aqueous solution, H_2_S is dissolved into HS^−^ and S^2−^. In acid solution, sulfide is chiefly in the form of H_2_S, while in the intermediate pH range (up to approximately pH 12), almost all the sulfide is in the form HS^−^. Only in very basic does the sulfide exist primarily as free ion (S^2−^). The SISE from Thermo Scientific supplies sulfide antioxidant buffer could maintain a fixed level of H_2_S.

Nevertheless, the alkaline condition of antioxidant buffer is regarded as an influencing factor to SISE measurements in plasma. Initially, mixing samples to antioxidant buffer is reported to generate protein desulfuration and artificially increased sulfide values [72]. It is also observed that placing 5% bovine serum albumin into antioxidant buffer leads to a surging reading of total sulfide measured by SISE in the first 20 minutes and following slow accumulation in 3 hours [73].

### 5.3. Fluorescent Probe Assays

Currently, there are more and more labs that choose to use fluorescent probes to assay the concentrations of real-time H_2_S, sensitively, selectively, and biologically compatible. There are 3 types of fluorescent probes for H_2_S detections: reduction-based, nucleophilic-based, and metal sulfide-based.

Reaction-based fluorescent probes for H_2_S detection are designed based on the reducing ability of H_2_S [74]. The firstly developed fluorescent probes by Lippert and colleagues were probes SF1 and SF2 based on the H_2_S-mediated reduction from an aryl azide to an aryl amine [75]. After adding NaHS for 1 hour, probes SF1 and SF2 detected 7- and 9-fold fluorescent increase, respectively. Probes SF4–7 were improved by the same lab with enhanced sensitivity and cellular retention [76]. The group of Peng and colleagues simultaneously reported another fluorescent probe DNS-Az through the reduction of a sulfonyl azide to a sulfonamide with faster kinetics than aryl azide reduction but less adaption [77]. Later, various fluorophores were developed for H_2_S measurement with different colors and targeting specific organelles. Fluorescent probes SHS-M1 and SHS-M2 were reported by Bae et al. to detect mitochondrial moiety by incorporating triphenylphosphonium group [78]. SulpHensor by Yang et al. was designed to detect lysosome moiety due to the morpholine group [79]. AzMC was reported by Thorson et al. to screen CBS based on coumarin [80]. Other functional groups that can be reduced by H_2_S were utilized in the design of fluorescent probes, like nitro group. Montoya and Pluth reported the fluorescent probe HSN-1, which incorporates a nitro group into the 1,8-naphthalimide scaffold, but with greater thiol cross-reactivity than azide probes [81]. This weakness was attenuated by Wang et al. that increased electron-rich aromatic system on the nitro-based probe [82]. The concept of H_2_S-mediated reduction was extended to other fluorophore scaffolds by several laboratories [83–85].

Nucleophilic-based fluorescent probe for H_2_S detection is designed based on the strong nucleophilic HS^−^ hydrolyzed from H_2_S at physiological pH (pH = 7.4) [86]. Qian et al. used this concept to develop fluorescent probes, SFP-1 and SFP-2, which allowed fluorescence switching via HS^−^ addition to aldehyde and underwent an intermolecular Michael addition to unsaturated acrylate ester to form a thioacetal, producing stable tetrahydrothiophene with strong fluorescence [87]. Qian et al. designed the probes with an aldehyde group ortho to an *α*,*β*-unsaturated acrylate methyl ester on an aryl ring, which trapped H_2_S and modulated a fluorescence response through decreased photoinduced electron transfer (PET) quenching of the product [87]. Disulfide bond cleaved by H_2_S was utilized by Liu et al. and Peng et al. to develop WSP1–5, which persulfide group, like 2-thiopyridine, intramolecular nucleophilic attacked on the ester moiety to release great fluorophore [88, 89]. 50–500 *μ*M H_2_S in bovine plasma and 250 *μ*M H_2_S in cells could be detected by this probe. Reversible nucleophilic addition was exploited by Chen et al., as CouMC, to track real-time H_2_S fluxes due to fast and potentially reversible fluorescence [90].

Metal sulfide-based fluorescent probe for H_2_S detection is based on the phenomenon that heavy metal ions such as Fe^3+^ and Cu^2+^ quench the fluorescence of a nearby fluorophore [91]. Zinc sulfide complex was utilized to design a selective fluorescent probe of H_2_S by Galardon et al. by releasing a coumarin dye [92]. Choi chose copper sulfide precipitation to design the fluorescent sulfide sensor [93]. Later, Sasakura et al. developed it to HSip-1, which possessed a cyclen macrocycle with fluorescein and binds Cu^2+^ to release unbound cyclen-AF, displaying greater fluorescence [94]. The measuring range of this probe for sulfide could be 10–100 *μ*M. Hou et al. improved the copper-containing probe to a lower detection limit of 1.7 *μ*M [95]. Another strength of metal precipitation-based probes is that they respond to turn on within seconds, allowing the real-time H_2_S detection [96]. Researchers may choose one of these fluorescent probes depended on their facilities, reagents, targeted organelles, and sensitivity ranges.

### 5.4. Other Analyzing Methods

Carbon nanotube (CNT) was introduced by Wu et al. for measuring low-concentration and nanoquantity H_2_S [97, 98]. One of the benefits of unfuctionalized CNT in analyzing H_2_S is due to the special bond with H_2_S, but other proteins kept in serum. H_2_S concentrations are reflected by the intensity of the fluorescence of the unfuctionalized CNT, due to the two values in a linear relationship. The lowest H_2_S concentration that can be tested is 20 *μ*M and smallest quantity of H_2_S is 0.5 *μ*g. The series of experiments are trying to establish a new sensor to measure micro- or nanoquantity H_2_S, comprising unfuctionalized CNT as a transducer and LSM fluorescence as a signal acquisition modality.

Polarography is a voltammetric measurement which makes use of the dropping mercury electrode or the static mercury drop electrode. The value of diffusion current depends on the speed of electroactive material (samples) diffusing to dropping mercury electrode. This principle contributes to the measurement of the concentration of analytes. Polarography is well known for the application of quantitative measurements of O_2_ (polarographic oxygen sensor, POS) and NO (polarographic nitric oxide sensor, PNOS). By recent years of the appreciation of the third gasotransmitter, H_2_S, several analytical methods are utilized, including polarography. A novel polarographic hydrogen sulfide sensor (PHSS) has been developed for the study of H_2_S-producing rates and consumption in mammalian tissues, with resolution of 10 nM [99]. The polarographic sulfide sensor is also applied to the investigation of kinetics of sulfide metabolism in organisms living in sulfide-rich environment [100]. PHSS permits direct and simultaneous measurement of H_2_S gas in biological fluids without sample preparation. PHSS has provided an alternative method for sulfide measurement.

Gas chromatography is a recent method described by Levitt et al. as a unique chemiluminescence-based technique to measure free and acid-labile H_2_S in multiple tissues from mouse [101]. The tissues were first submerged in 50 mM glycine-NaOH buffer (pH 9.3) and homogenized. The homogenates were then transferred to syringes, which were sealed and flushed with N_2_. The homogenate in alkaline extraction turns to acidification to pH 5.8 by adding sodium hydrogen phosphate solution (pH 5.5). After vigorous mixture, the gas space was removed to gas chromatography to analyze free H_2_S concentration. Next, adding 50% trichloroacetic acid to the syringe, the gas was collected to test the acid-labile H_2_S concentration. The flow rate of N_2_ was 25 ml/min. The concentration of H_2_S was calculated by the plotting H_2_S standard curve.

**Figure 5:**
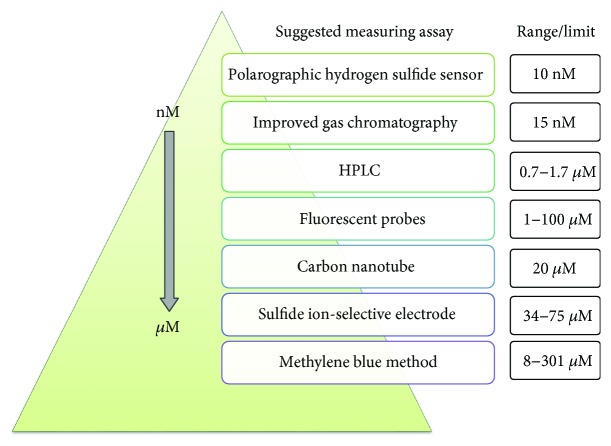
The ranges or limits of H_2_S measurements.

High-performance liquid chromatography (HPLC) is used to separate the sulfide mixture. Togawa et al. reported that using monobromobimane (MBB) with dithiothreitol (DTT) reacted with bound sulfide to produce sulfide dibimane, which is separated from MBB by HPLC and detected by its fluorescent probes [102]. Recently, MBB assay without DTT was used to measure available H_2_S in rat blood [103] and mouse plasma [104]. The ranges or limits of H_2_S measurements are in Figure 5.

## 6. H_2_S in Inflammation

Inflammation is an immune response to an injury or harmful stimuli, in order to self-protect the body from avoiding pathogen assaults and initiating healing process. However, the adaptive immune system fails to counter invading agents will turn to target host tissues, making deeply more serious damage. H_2_S regulating inflammation and injury was initially contradictory, but in recent years, more studies supported that H_2_S inhibited the process of inflammation, except at high concentration [105]. This mediator possibly exerts its anti-inflammatory effects through reduction of leukocyte-endothelial cell adhesion [106], action on ATP-sensitive K^+^ channels [107], scavenging of toxic free radicals [108], elevation of cyclic AMP and/or cyclic GMP [70, 71], and inhibition of nuclear factor-*κ*B (NF-*κ*B) and proinflammatory cytokines (e.g., COX-2 [109], iNOS [110], and interleukin- (IL-) 1*β*, IL-6 [111]).

Various diseases could be found inflammatory response, like atherosclerosis, ischemia-reperfusion, and colitis. Contributing to anti-inflammatory molecular mechanisms of this novel gasotransmitter, it is not surprising that H_2_S may participate in the process of resolution of a variety of inflammatory diseases. In atherosclerosis, H_2_S exerts its potent inhibitor of leukocyte adherence to vascular endothelium [112]. Meanwhile, the generation of reactive oxygen species (ROS), activation of NF-*κ*B, increased expressions of cell adhesion cytokines, and induction of apoptosis, which were all regarded as the key promoters of pathology, were all found suppressed by H_2_S [112, 113]. These mechanisms of action described for H_2_S may explain that H_2_S can diminish the plaques in arteries and attenuate the atherosclerotic injury, suggesting the character of anti-inflammation of H_2_S is a benefit for the vascular protection.

Ischemia-reperfusion (I/R) is identified as an acute endogenous inflammatory response that characterizes release of toxic free radicals, leucocyte-endothelial cell adhesion, and platelet-leucocyte aggregation [114]. In porcine myocardial I/R model, therapeutic sulfide improved myocardial function and diminished infarct size though decreased levels of inflammatory cytokines (IL-6, IL-8, and TNF-*α*), reduced left ventricular pressure, and improved coronary microvascular reactivity [115]. A similar tissue protection of H_2_S was also found in hepatic I/R injury by inhibition of inflammation (lipid peroxidation, IL-10, ICAM-1, and TNF-*α*) and apoptosis (caspase-3, Fas, and Fas ligand) [116]. Another study suggested that the cardioprotective effects of H_2_S may be mediated by opening the mitochondrial K_ATP_ channel and second window of protection caused by endotoxin [117].

Colitis is a one form of gastrointestinal inflammation and ulceration. Administration of H_2_S-generating agents or precursor for H_2_S synthesis, L-cysteine, has been shown to significantly accelerate ulcer healing [118, 119]. This ability of H_2_S to enhance gastrointestinal resistance attracts investigators to exploit novel treatments of gastrointestinal injury and inflammation, like H_2_S-releasing derivative of NSAIDs to reduce the adverse drug reaction of NASIDs, retarding gastrointestinal ulcer healing [120]. Evidence of H_2_S in resolution of colitis in rats or mice studies showed that administration of H_2_S donor significantly inhibited the severity of colitis with marked reduction of granulocyte infiltration into colonic tissue. In inflamed colon, H_2_S production was highly increased via CSE, CBS, or other enzymatic pathways [121, 122]. Once H_2_S synthesis was inhibited, the colitis tended to worsen the inflammation with thickening of the smooth muscle, perforation of bowel wall, and even death [110].

## 7. H_2_S in Redox Status

### 7.1. H_2_S Direct Effects on Toxic Free Radicals

In a weak acid, H_2_S dissociates in equilibrium with hydrosulfide anion (HS^−^) and sulfide anion (S^2−^). Under physiological conditions, the amounts of H_2_S and HS^−^ are early equal within the cell, whereas extracellular fluid and plasma exist approximately the ratio of 20% H_2_S, 80% HS^−^, and 0% S^2−^. HS^−^ is a potent one-electron reductant that eliminates free radicals by donating single electron. Hydrogen disulfide (H_2_S_2_), a kind of hydrogen polysulfide (H_2_Sn), is the production of oxidation of HS^−^ by two-electron oxidants, like hypochlorous acid [123] and hydrogen peroxide [124]. Additionally, the chemical interaction between H_2_S and NO also produced H_2_Sn by activating transient receptor potential ankyrin 1 (TRPA1) channels [36]. H_2_S_2_, a highly reactive oxidizing chemical, generates H_2_S by reacting with thiol [125] or disproportionation [123, 126]. H_2_S_2_ and H_2_S_3_ were reported to generate redox regulators Cys-SSH and GSSH via 3MST in the brain of wild-type mice but not in those of 3MST-KO mice [34, 35, 127, 128].

H_2_S is considered as an endogenous reducing agent which is produced in response to oxidative stress [129, 130]. Evidence showed that H_2_S is a highly reactive molecule and may easily react with other compounds, especially with reactive oxygen and nitrogen species. H_2_S reacts with at least four different ROS: superoxide radical anion [69], hydrogen peroxide [67], peroxynitrite [70], and hypochlorite [71]. All these compounds are highly reactive, and their reactions with H_2_S result in the protection of proteins and lipids against RNS/RNS-mediated damage [70, 71] and myocardial injury induced by homocysteine in rats [131].

### 7.2. H_2_S Protects Mitochondria against Oxidative Stress

Mitochondrial injury is an important source of reactive oxygen species (ROS), which is involved in a range of pathologies, such as ischemia-reperfusion, atherosclerosis, and toxin exposure [132]. Under oxidative stress conditions, mitochondria will show unstable mitochondrial membrane potential (Δ*Ψm*), redox transitions, and negative changes in the mitochondrial permeability transition (MPT) pore and the inner membrane anion channel (IMAC) [133]. Our lab found that H_2_S can reduce the H_2_O_2_-induced injury in HUVECs via increasing ATP production, saving mitochondrial ultrastructure, stabilizing mitochondrial membrane intact, decreasing ROS and MDA, and rising antioxidants. The same situation was also unveiled in H_2_O_2_-stimulated isolated rabbit aorta that H_2_S ameliorated mitochondrial dysfunction through improving O_2_ consumption and ATP production, protecting mitochondrial respiration chain complexes activities and matrix enzymes, decreasing mitochondrial membrane permeability, and inhibiting mitochondrial ROS levels. These effects of H_2_S indicated that the antioxidative ability of H_2_S is through increasing antioxidants and prohibiting ROS levels and also preserving mitochondrial function to reduce the production of toxic free radicals.

## 8. H_2_S in Cardiovascular System

### 8.1. Hypertension

Before identified as the third gasotransmitter, H_2_S has been speculated to regulate an array of physiological processes in regulating cardiovascular functions, distinctive from its toxicological effect. A great number of studies have been carried on investigation of the modulating of blood pressure by exogenous and endogenous H_2_S. Early at the end of the last century, it is first reported that H_2_S relaxes the contracted smooth muscles (SM) induced by 1 *μ*M norepinephrine in rat thoracic aorta and portal vein [134]. The relaxations in these tested aortas and veins present a NaHS dose-dependent manner, but the potency of relaxation by exogenous H_2_S in the thoracic aorta is less than the portal vein, even by 10^−3^ M NaHS, which are around 25% and 90%, respectively. The data also showed that the relaxation effects of H_2_S and NO can be enhanced by each other. 30 *μ*M NaHS can augment the loosening effect of NO by up to 13-fold. Thus, endogenous cysteine and glutathione do not have synergistic effect with NO. Subsequently, the vasorelaxant effect of H_2_S was found *in vivo* of SD rats, ex vivo of aortic rings, and *in vitro* at rat aortic smooth muscle cells [15], which was a literature that first demonstrated the underlying mechanism of vasorelaxation, a consequence of opening K_ATP_^+^ channels. Interestingly, it has been found that H_2_S induces endothelium-dependent vasorelaxation with many common mechanistic traits of hyperpolarizing factor [135]. CSE knockout mice lacked the methacholine-induced endothelium-dependent vasorelaxation in mesenteric arteries and showed higher resting membrane potential of SMCs, while hyperpolarization of SMCs induced by methacholine was observed in endothelium-intact mesenteric arteries at wild-type mice [136]. Administration of exogenous H_2_S hyperpolarized both SMCs and vascular endothelial cells in wild-type and CSE knockout mice [136]. Removal of functional endothelium attenuated vasorelaxation of rat aorta [137] and rat mesenteric artery [138]. It appears that vasorelaxation of H_2_S is induced on both SMCs and endothelial cells, instead of previous research discussions mainly focusing on SMCs.

A multitude of H_2_S-induced vasodilation studies have investigated the activation of K_ATP_^+^ channels. One possible mechanism involved in the activation of K_ATP_^+^ channels by H_2_S was opening K_ATP_^+^ channels and increasing K^+^ currents resulted in hyperpolarizing membrane of smooth muscle cells [139]. The explanation of the opening of K_ATP_^+^ channels by H_2_S was that cysteines on K_ATP_^+^ channels of SMCs were S-sulfhydrated, leading to hyperpolarization [140]. Cys43 of the inwardly rectifier (Kir) potassium channels subunit Kir 6.1 was sulfhydrated by NaHS, eliciting the binding to phospholipid phosphatidylinositol (4,5)-bisphosphate (PIP2) together with decreased association of ATP [140]. Additionally, the vasodilation effect of H_2_S was inhibited significantly by either using a calcium-free bath solution or with the normal bath solution, but in the presence of nifedipine, a voltage-gated Ca^2+^ channel inhibitor, on aortic rings [8], indicates that the vascular effects of H_2_S are also likely mediated by the attenuation of intracellular inward Ca^2+^ currents. Not only H_2_S hyperpolarizes ion channels on blood vessels to possess the relaxant effects but also endothelium generates H_2_S by increasing catalytic activity of CSE through calcium-calmodulin, indicating that the H_2_S formation may be involved in vascular activation to reduce blood pressure [141]. Moreover, H_2_S exerts cardioprotective effect by relieving vascular structural remodeling observed during hypertension, including suppression of VSMC proliferation via the activation of cardiac extracellular signal-regulated kinase (ERK) and/or Akt pathway [137] and attenuation of collagen accumulation through reduction of collagen type I level, [3H] thymidine and [3H] proline incorporation, and [3H] hydroxyproline secretion in the SHRs [142] and through mitrogen-activated protein kinase (MAPK) pathway [143]. As endothelium-derived relaxing factors (EDRF), H_2_S and NO have “cross-talk” on the calcium mobilization [144], activation of eNOS [145–148], PI3K/Akt signaling [145], soluble guanylate cyclase (sGC) [145, 149], and cGMP [150, 151]. However, whether NO is directly involved in the antihypertensive effects of H_2_S has to be further investigated by a NO deficiency model induced to hypertension and treated by sulfide-rich compounds.

### 8.2. Atherosclerosis

Atherosclerosis is a chronic and slowly progressive cardiovascular disease that affects arterial blood vessels by thickening and hardening as consequences of the high plasma cholesterol concentrations, especially cholesterol in low-density lipoprotein [152]. Cholesterol deposition, lipid oxidization, cell adhesion, vascular inflammation, foam cell accumulation, smooth muscle cell migration, and plaque calcification are involved in different stages of the pathological process [153]. The cumulative plaques consequentially narrow the arterial lumen and restrict blood supply. Severe atherosclerotic lesions are the high risk factors of ischemic diseases such as stroke and heart attack [154].

Recent years, H_2_S draws attentions from researchers by its cardiovascular protective effects, while there are not many studies on its effects on the progress of atherosclerosis. Fortunately, increasing evidence has indicated that H_2_S plays a potentially significant role in a number of biological processes and potential cardiovascular protections, which suggest that H_2_S may contribute to the inhibition of pathogenesis of atherosclerosis. First, H_2_S shows inhibitory effects on the development of atherogenesis, such as oxidative stress, modificated oxidation of LDL, cell adhesion, and calcification. In vascular smooth muscle cells (SMCs), low levels of NaHS (30 or 50 *μ*M), a donor of H_2_S, decrease toxic reactive oxygen species, including H_2_O_2_^−^, ONOO^−^, and O_2_^−^ [155]. At the same time, NaHS also enhances the functions of antioxidative enzymes. In addition, H_2_S inhibits atherogenic modification of LDL-induced HOCl in vitro (such as oxidized LDL, shortened as oxLDL). As a potent atherogenic agent, oxLDL particle is an important product of atherogenic oxidation that stimulates endothelial cells to express various adhesion molecules for consequent inflammatory reactions and formation of foam cells. Therefore, inhibition of oxLDL by potential treatments of H_2_S implies that H_2_S may interfere atherosclerotic progress [156]. Furthermore, H_2_S attenuates atherosclerotic lesions by reducing cell adhesion molecules, such as ICAM-1, involving the NF-*κ*B pathway *in vivo* and *in vitro* [112]. Adhesion molecules are the significant causes to promote bindings between monocytes and T lymphocytes to endothelial cells, which will lead to sequential inflammation and advanced process. Reduced expressions of adhesion molecules prohibit monocytes migration and later inflammation, which may also benefit in ameliorate atherosclerotic lesions. Lastly, calcification, presented in the advanced process of atherosclerosis, is a potent factor of plaque stability. There was a study that found the link between H_2_S and plaque calcification [157]. In calcified arteries, H_2_S level, CSE activity, and CSE mRNA were downregulated, while after administration of H_2_S, a dose response was shown in the decreased vascular calcium content, Ca^2+^ accumulation, alkaline phosphatase (ALP) activity, and aortic osteopontin (OPN) mRNA. These changes speculated the effect on atherogenesis of H_2_S might be induced by suppressing vessel calcification.

Second, H_2_S possesses vascular protective capacities from inhibition of proliferation of vascular cells, such as intima and SMCs, and angiosteosis. It has been demonstrated that H_2_S suppresses neointima hyperplasia on rat carotid after balloon injury [158]. In another balloon-injured artery experiment, NaHS (30 *μ*mol/kg bodyweight) enhances methacholine-induced vasorelaxation and significantly ameliorates neointimal lesion formation. Additionally, evidences are also pointing to the fact that H_2_S relieves apoptosis and proliferation of SMCs [159]. SMCs migrate from the medial layer into the subendothelial space where they may proliferate, ingest modified lipoproteins, secrete extracellular matrix proteins, and contribute to lesion development. The suppression of proliferation of SMCs by H_2_S can restrict atherosclerotic damages. Moreover, H_2_S prevents the process of angiosteosis [143, 160, 161]. Angiosteosis, ossification or calcification of a vessel, is an advanced change in the pathology of atherosclerosis. Its development leads to the narrowing of the caliber of an artery, stimulates thrombosis, or even worse generates the abruption of unstable plaques. Vascular calcifications induced by vitamin D_3_ and nicotine in rats are ameliorated by exogenous H_2_S. The responses after administration of H_2_S show the decreased calcium concentration in vessels, reduced expressions of angiosteosis, and accompanied acidic phosphatese and osteopontin.

Third, H_2_S alleviates the vascular damage induced by an established risk factor, for instance, homocysteine. Homocysteine is an amino acid, biosynthesized from methionine and converted into cysteine and sulfur. Augmented levels of homocysteine in plasma, termed hyperhomocysteinemia, are considered as a high risk factor of atherogenesis. Early plaque development in apolipoprotein E-deficient mice, a knockout genetic model of atherosclerosis by 8 weeks high-cholesterol diet intake, could be enhanced by dietary supplementation with methionine or homocysteine [162]. A research shows that low concentrations of NaHS (30 or 50 *μ*M), a H_2_S donor, potentiates cell viability of rat aortic SMCs by abating cytotoxicity and reactive oxygen species stimulated by hyperhomocysteinemia [163].

Although atherosclerosis is a chronic, systemic disease with multifactors involved in its initiation and progression, previous studies have shown that the specific characteristics and functions of H_2_S may contribute to the inhibition of atherogenesis. The multiaspect recognitions of cardiovascular protective effects of H_2_S provide a new avenue of antagonism towards this complicated cardiovascular disease.

### 8.3. Myocardial Injury

Plenty of work have documented that the CSE/H_2_S pathway participates in the regulation of cardioprotective effects [155]. Administration of exogenous H_2_S reduces “infarct-like” myocardial necrosis induced by isoproterenol in the rat [67, 164, 165]. This protection is accompanied with the reduced concentrations of H_2_S in myocardium and plasma, decreased CSE protein activity, and upregulated CSE gene expression in myocardium [67]. NaHS attenuates the myocardial ischemic injury by evidences of reduced mortality and shrunk infarct size in vivo of rat and recovered SMC viability induced by hypoxia [67]. Further study discovers that 14 *μ*mol/kg/d NaHS improves ECG and blood pressure and diminishes infarct size, as well as the greater survivin expression [165].

Oxidative stress injury is an important mechanism of myocardial injury. Direct or indirect antioxidative effects will lead to cardioprotection from myocardial ischemia. The data in above literature reveal that NaHS may antagonize MDA production *in vitro* of myocytes by oxygen free radicals or directly react with hydrogen peroxide and superoxide anions [166]. Another experiment also proves that H_2_S provided profound protection against ischemic injury by significant decreases in infarct size, circulating troponin I levels, and oxidative stress [67]. The protections by Na_2_S in early and late preconditioning are all though stimulating the increased antioxidants, which could be itemized to the elevated Nrf2 in early stage and increased expressions of heme oxygenase-1 and thioredoxin 1 in late preconditioning. The antioxidant effect of H_2_S is also embodied in the preservation of mitochondrial functions and ultrastructure by Na_2_S after myocardial ischemia-reperfusion (MI-R) injury [167]. These observations have been recently confirmed by cysteine analogues, SAC, SPC, and SPRC [168, 169]. The activities of superoxide dismutase (SOD), catalase (CAT), glutathione peroxidase (GPx), and glutathione redox status are preserved by cysteine analogues. The mitochondrial ultrastructure of cysteine analogues treatments appeared more normal than MI vehicle group. These evidences demonstrate the CSE/H_2_S pathway is involved in reducing the deleterious effects of oxidative stress.

Furthermore, recent discoveries indicate the observed protection of H_2_S is related to regulate leukocyte adhesion and leukocyte-mediated inflammation, increase anti-inflammatory cytokines, and reduce several proinflammatory cytokines [169]. The anti-inflammatory effect of H_2_S is reflected in amplification of heat shock protein (HSP) 70, HSP 90, and cyclooxygenase-2 [115] and reduction of MPO activity [167], nuclear factor-*κ*B (NF-*κ*B), and interleukin (IL)-6, IL-8, and tumor necrosis factor-alpha (TNF-*α*) [167]. The cardioprotection of H_2_S is associated with inhibition of cardiomyocyte apoptosis after myocardial injury. H_2_S amplifies antiapoptosis proteins (Bcl-2, Bcl-xL) and inactivates proapoptogen (Bad) [115]. It is also suggested that H_2_S ameliorates cardiomyocyte apoptosis after MI-R injury *in vitro* and *in vivo*, significant abatement of caspase-3 activity, and declining of the number of TUNEL positive nuclei, respectively [167].

Finally, multiple studies have elucidated a protective effect of K_ATP_ channel activators in myocardial MI-R injury [168]. By virtue of the relaxant effect of H_2_S as an opener of K_ATP_ channels, it is easy to hypothesize that H_2_S protects myocardial cells against ischemic injury. In the isolated Langendorff-perfused rat hearts, administrations of NaHS result in a dose-dependent limitation of infarct size induced by left coronary artery ligation and reperfusion, while this protective effect is abolished by K_ATP_ channel blockers [170]. There is a report that H_2_S preconditioning presents cardioprotective effects against ischemia though signaling pathways of K_ATP_/PKC/ERK1/2 and PI3K/Akt [171]. Researchers may investigate additional molecular mechanisms to explain this ischemic injury in hearts not limited on stereotyped mechanisms, such as oxidative stress or potassium channels.

### 8.4. H_2_S in Angiogenesis

The term “angiogenesis” is referred to the physiological process of blood vessel growth or vessel sprouting [172]. Blood vessel growth can benefit for delivering nutrients and waste and supplying immune surveillance [172]. Insufficient vessel growth has been linked to stroke, myocardial infarction, ulcerative disorders, hair loss, preeclampsia, and neurodegeneration [173]. Embryonic development, menstrual cycle, hypoxia, inflammation, and tumor will stimulate angiogenic signals, such as vascular endothelial growth factor (VEGF), angiopoietin-2 (ANG-2), and fibroblast growth factors (FGFs) to sprout new endothelial cells and pericytes or vascular smooth muscle cells [173, 174].

H_2_S has been displayed as an important regulator of angiogenesis through promoting endothelial proliferation, migration, and formations of tub-like structure and networks. Administration of H_2_S increased proliferation and migration in bEnd3 microvascular endothelial cells and recovered microvessel sprouting in rat aortic rings of silencing CSE [145]. We discovered that SPRC, as a H_2_S donor, enhanced HUVEC cell proliferation, adhesion, migration, and tube formation as well as the same effects in the rat aortic ring and Matrigel plug models [175]. In vivo studies of mouse hindlimb ischemia and rat myocardial ischemia provided additional evidence that SPRC ameliorated ischemic insults through augmenting angiogenesis [175]. Considering H_2_S and NO share angiogenic effects, we synthesized H_2_S-NO hybrid molecule, named ZYZ-803, to slowly release H_2_S and NO [176]. As expected, ZYZ-803 presented significantly greater potency of angiogenesis than H_2_S and NO alone [176]. Besides CSE-mediated effects, some studies showed that RNAi-mediated silencing CBS leads to a 40–50% decrease in HUVEC proliferation and 30% decrease in tube length on Matrigel [177]. Using AOAA, the CBS inhibitor developed a dose-dependent decrease of HUVEC proliferation rate, indicating that CBS is also involved in mitogenic effects of H_2_S [177]. Supplying 3MP, the 3MST substrate, facilitated wound healing and reserved mitochondrial functions which were associated with greater proliferation rates, proven by silencing 3MST to inhibit ECs growth and migration rates [178]. Taken together, H_2_S may be a potential proangiogenic agent, which is independent of the three synthesizing enzymes.

To determine how H_2_S regulates endothelial functions, most studies focused on the VEGF (also called as vascular permeability factor, VPF) signaling, which is the arguably crucial pathway in angiogenic responses both under healthy and pathophysiological circumstances [173, 179]. Silencing CSE and CSE inhibitor PAG reduced vessel length and branching stimulated by VEGF [145, 180]. Meanwhile, incubation of VEGF in HUVECs resulted in higher H_2_S synthesis and level [180]. Additionally, H_2_S presented as an endogenous stimulator of angiogenesis by increasing the activation of Akt, ERK, and p38, which are the downstreams of VEGF signaling [180]. Administration of glibenclamide, the K_ATP_ channel blocker, reduced H_2_S-induced endothelial cells motility and prohibited H_2_S-triggered activation of p38, indicating K_ATP_ channel was one of the H_2_S targets and may locate at upstream of p38 in this motility process [180]. We first developed SPRC as the H_2_S donor which activated and interacted with signal transducer and activator of transcription 3 (STAT3) to induce angiogenesis *in vitro* and *in vivo* [175]. We also discovered that ZYZ-803, releasing H_2_S and NO, regulates angiogenesis through SIRT1/VEGF/cGMP pathway [176]. However, how the STAT3 links to Akt signaling, ERK/p38, and K_ATP_ channel still needs further investigations.

## 9. Conclusion and Perspectives

**Figure 6:**
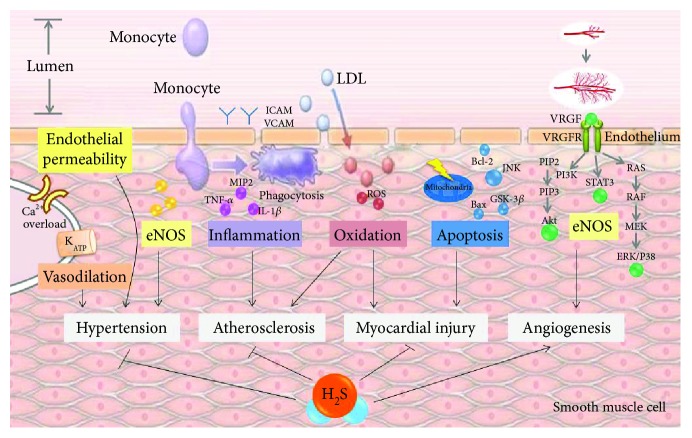
Schematic illustration of molecular mechanisms underlying H_2_S-induced cardioprotection.

Over the last few decades, there are significant progress achieved in delineating the therapeutic potentials and molecular mechanisms underlying the actions of H_2_S on cardiovascular diseases [181], seen in Figure 6. The evidences elaborated above indicate that H_2_S derived from CSE, CBS, 3MST/AAT, or DAO reduces blood pressure, inhibits atherosclerotic progress, alleviates infarct myocardial injuries, and stimulates the angiogenic properties on endothelium. Therefore, several chemicals have been developed to test the therapeutical potentials for further drug development in human. In spite of compelling evidences in the literature for the role of exogenous and endogenous and H_2_S in vessel and myocardial protection, several questions regarding to precise mechanisms and regulations of H_2_S in the context of cardiovascular diseases need to be better understand. In quiescent, growing, and maturing vessels, does the generation of H_2_S generated by different cell types have any interaction and which one plays the major role? Is the H_2_S-mediated inflammation different in high blood pressure, angiogenesis, ischemic injury, and atherosclerosis? What is the exact manner of cross-talk between the three gas neurotransmitters, that is, NO, CO, and H_2_S? Interestingly, some studies showed obvious discrepancy by suggesting vasoconstrictor effects of H_2_S, instead of vasodilation actions. Further studies will be required to determine whether this discrepancy is due to dose of H_2_S, vascular response, oxygen tension, or experimental models. Finally, the posttranslational level of H_2_S-producing enzymes should be defined in the context of regulations and activities. After these tremendous growths of preclinical studies, we expect the sulfide-containing compounds will apply to clinics someday with considerable efficacy and safety.

## Figures and Tables

**Table 1:** Comparison of nitric oxide, carbon monoxide, and hydrogen sulfide.

	Nitric oxide	Carbon monoxide	Hydrogen sulfide
Formula	NO	CO	H_2_S
Color and odor	Colorless; a mild, sweet odor	Colorless; odorless	Colorless; smell like rotten egg
Free radical	Yes	No	No
Flammable	No	No	Yes
Toxicity	Yes	Yes	Yes
Inhibition of mitochondrial cytochrome c oxidase	Yes	Yes	Yes
Resources	L-arginine or nitrite	Protohaem IX	L/D-cysteine
Intermediate products	L-NG hydroxyarginine, citrulline	Biliverdin IX-*α*	Cystathionine, L-cysteine, *α*-ketobutyrate, and pyruvate
Enzymes	eNOS, iNOS, and nNOS	HO-1, HO-2, and HO-3	CBS, CSE, 3MST/AAT, and DAO
Vascular effect	Vasodilation, angiogenesis	Vasodilation, angiogenesis	Vasodilation, angiogenesis
Inhibition inflammation	Yes	Yes	Yes
Antiapoptosis	Yes	Yes	Yes
Haem effect	Yes	Yes	Yes
Molecular targets	Soluble guanylate cyclase (sGC)	Soluble guanylate cyclase (sGC)	K_ATP_ (ATP-gated potassium) channel
Targeting outcome	Increase cGMP, activate K_Ca_ channels and nitrosylation	Increase cGMP, activate K_Ca_ channels	Increase cGMP and cAMP, activate K_ATP_ channels and sulfhydration
Application on human	Pulmonary hypertension, lung transplantation, and ARDS	Not available	Not available

**Table 2:** Characteristics of H_2_S-producing enzymes.

	Cystathionine *γ*-lyase (CSE)	Cystathionine *β*-synthase (CBS)
Localization	Liver, heart, vessels, kidney, brain, adipose, small intestine, stomach, uterus, placenta, and pancreatic islets	Brain, liver, kidney and ileum, uterus, placenta, and pancreatic islets
Activators	Pyridoxal 5′-phosphate	Pyridoxal 5′-phosphate, S-adenosyl-L-methionine, and Ca^2+^/calmodulin
Inhibitors	D,L-propargylglycine, *β*-cyano-L-alanine	Hydroxylamine, aminooxyacetate
Functional roles	H_2_S production in the liver and smooth muscle	H_2_S production in the brain and nervous system

**Table 3:** H_2_S-releasing compounds used in basic scientific researches.

Compounds	Constituents	Effects on research fields
SAC	S-allylcysteine	Protection on cardiovascular and neural systems
SPRC	S-propargyl-cysteine	Anticancer, anti-inflammation, and antihypoxic/ischemia and impairs cognition and A*β*-induced neuronal damage
GYY4137	Morpholin-4-ium-4-methoxyphenyl (morpholino) phosphinodithioate	Antagonizes endotoxic shock though anti-inflammatory effects
ACS-6	A H_2_S-donating sildenafil	Inhibits superoxide formation and gp91^phox^ expression in porcine PAECs
ACS-14	A H_2_S-releasing aspirin	Regulates redox imbalance, such as GSH formation, HO-1 promoter activity, and isoprostane suppression
ACS-15	A H_2_S-releasing derivative of diclofenac	Arthritis
ACS-67	A H_2_S-releasing derivative of latanoprost acid	Glaucoma; retinal ischemia
ATB-284	A H_2_S-releasing derivative of trimebutine	Irritable bowel syndrome
ATB-337	A H_2_S-releasing derivative of diclofenac	Gastrointestinal damage induced by NSAIDs
ATB-346	A H_2_S-releasing derivative of naproxen	Acute and chronic joint pain
ATB-429	A H_2_S-releasing derivative of mesalamine	Inflammatory bowel disease and antinociceptive and anti-inflammatory effects
IK 1001	Calcium-cross-linked alginate polymer	Suspended animation, multiple hypoxic/ischemic conditions, cardiac remodeling, and congestive heart failure
